# Metabolic Programming of Tumor-Associated Macrophages in Head and Neck Squamous Cell Carcinoma: Implications for Innate Immunity and Therapeutic Response

**DOI:** 10.3390/biology15070561

**Published:** 2026-03-31

**Authors:** Vincent G. Yuan

**Affiliations:** Department of Otolaryngology-Head and Neck Surgery, University of Pittsburgh Medical Center, Pittsburgh, PA 15213, USA; vincentyuan@pitt.edu

**Keywords:** tumor-associated macrophages, head and neck squamous cell carcinoma, metabolic reprogramming, innate immunity, hypoxia, lactate, immunometabolism, therapeutic resistance

## Abstract

Head and neck squamous cell carcinoma (HNSCC) develops within a chronically inflamed and metabolically constrained tumor microenvironment that profoundly shapes innate immune function. Tumor-associated macrophages (TAMs) are abundant in HNSCC and display marked plasticity, yet predominantly acquire immunosuppressive states that promote tumor progression and therapeutic resistance. Traditional M1/M2 polarization models fail to capture this dynamic behavior. Emerging evidence instead identifies metabolic reprogramming as a central regulator of macrophage function in cancer. Hypoxia, nutrient limitation, extracellular acidification, and tumor-derived metabolites such as lactate and lipids rewire macrophage metabolism, directly influencing inflammatory signaling and immune suppression. This review integrates recent advances in immunometabolism to examine how metabolic adaptations govern macrophage innate functions in HNSCC and highlights therapeutic opportunities targeting macrophage metabolism to overcome immune resistance.

## Simple Summary

Head and neck cancer remains a serious disease with limited treatment success for many patients. A key reason is the tumor environment, which can weaken the body’s natural defenses. This review focuses on a type of immune cell called macrophages, which normally help fight disease but can be altered by tumors to support cancer growth. We explain how harsh conditions inside tumors, such as low oxygen, limited nutrients, and the buildup of waste products, change how these cells function. These changes affect how macrophages produce signals, remove harmful cells, and interact with other parts of the immune system. Instead of protecting the body, they often suppress immune responses and help tumors survive and spread. Importantly, we highlight that these changes are driven by shifts in how macrophages use energy and nutrients. Understanding these processes opens new opportunities for treatment. By targeting these altered metabolic pathways, it may be possible to restore the ability of macrophages to fight cancer and improve the effectiveness of existing therapies. This work provides a clearer framework for developing new strategies to treat patients with head and neck cancer.

## 1. Introduction

Head and neck squamous cell carcinoma (HNSCC) represents a major global health burden, with high rates of morbidity and mortality despite advances in surgery, chemoradiotherapy, and immunotherapy [1]. HNSCC accounts for approximately 890,000 new cases and 450,000 deaths annually worldwide, underscoring its substantial clinical and socioeconomic burden. Major risk factors include tobacco use, excessive alcohol consumption, and infection with high-risk human papillomavirus (HPV), which define biologically and clinically distinct disease subsets [2]. The tumor microenvironment (TME) of HNSCC is highly heterogeneous and characterized by chronic inflammatory processes that shape tumor progression and responses to therapeutic intervention [3]. Innate immunity, the first line of defense against malignant transformation, plays a central role in this milieu, not only during early tumorigenesis but also throughout sustained interactions between malignant cells and stromal components [4].

Among innate immune populations, macrophages are particularly abundant within the HNSCC TME and exhibit pronounced functional plasticity that influences disease outcomes [5]. Tumor-associated macrophages (TAMs) can exert both anti-tumor and pro-tumor activities; however, in HNSCC, they predominantly adopt immunosuppressive, pro-tumor states that promote immune evasion, angiogenesis, invasion, and metastasis [6,7]. Consistent with this, high infiltration of CD163^+^ macrophages correlates with advanced tumor stage and poorer prognosis in HNSCC patients, and these cells often form structural barriers that limit effective immune infiltration following PD-1 blockade therapy [8,9,10].

While macrophages have traditionally been classified along an M1/M2 spectrum, this framework fails to capture their dynamic, context-dependent functions in cancer [11,12]. Increasing evidence indicates that metabolic reprogramming, defined as adaptive alterations in metabolic pathways that support cellular survival and function under adverse conditions, serves as a key regulator of macrophage behavior within the TME [13,14]. In response to hypoxia, nutrient scarcity, and extracellular acidification, immune cells remodel their metabolic programs, with direct consequences for their effector functions and immunoregulatory capacity [15].

Recent advances in cancer immunometabolism have demonstrated that metabolic pathways do more than satisfy bioenergetic demands; they actively shape macrophage polarization, cytokine production, and immunosuppressive signaling [16,17]. Beyond canonical metabolic circuits, emerging studies further reveal that specific metabolic enzymes and metabolites function as signaling nodes that regulate macrophage states, highlighting new opportunities for therapeutic intervention [18].

In HNSCC, although interactions between tumor cell metabolism and immune regulation have received increasing attention, the metabolic determinants governing macrophage innate functions remain under-reviewed and poorly integrated into prevailing models of chronic tumor-associated inflammation. Immunometabolic crosstalk between HNSCC cells and macrophages, mediated by lactate accumulation, lipid-derived signals, and hypoxia-driven pathways, contributes to TAM polarization and suppresses effective antitumor immunity [19].

Accordingly, this review synthesizes the current understanding of innate immunity in HNSCC with a focused emphasis on macrophage metabolism. We highlight how metabolic reprogramming shapes macrophage functional states, sustains chronic inflammatory pathophysiology, and contributes to therapeutic resistance, proposing integrative mechanistic frameworks that extend beyond classical immunophenotyping.

## 2. Macrophage Origins and Innate Features in HNSCC

### 2.1. Ontogeny: Bone Marrow-Derived Versus Tissue-Resident Macrophages

**Figure 1:**
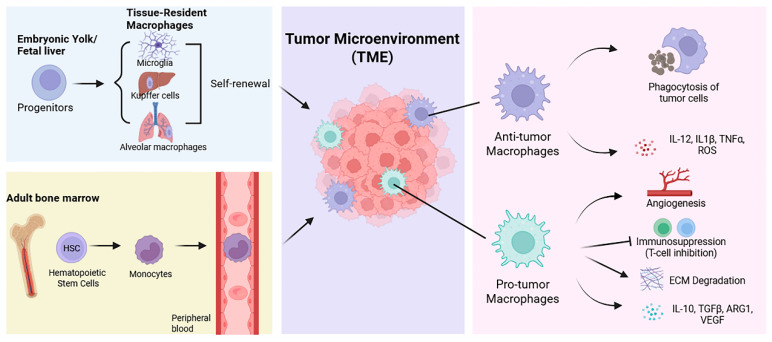
Ontogeny and Functional Specialization of Macrophages in the HNSCC Microenvironment. TAMs in HNSCC originate from distinct developmental lineages, including embryonically derived tissue-resident macrophages (e.g., microglia, Kupffer cells, and alveolar macrophages) that are maintained through local self-renewal, as well as adult bone marrow-derived HSCs that give rise to circulating monocytes infiltrating the tumor from the peripheral blood. Upon entry into the heterogeneous TME, these macrophages undergo extensive functional reprogramming and adopt divergent states. Anti-tumor macrophages promote host defense through tumor cell phagocytosis and the production of pro-inflammatory mediators such as Interleukin-12 (IL)-12, IL-1β, Tumor necrosis factor-alpha (TNFα), and reactive oxygen species (ROS). In contrast, pro-tumor macrophages facilitate disease progression by promoting angiogenesis, suppressing T-cell activity, driving extracellular matrix (ECM) remodeling, and secreting immunosuppressive factors including IL-10, transforming growth factor-beta (TGFβ), arginase-1 (ARG1), and vascular endothelial growth factor (VEGF).

Macrophages within tissues arise from two principal developmental origins: circulating monocytes derived from bone marrow hematopoietic stem cells (HSCs) and tissue-resident macrophages seeded during embryogenesis (Figure 1) [20]. The classical mononuclear phagocyte system proposed that tissue macrophages are continuously replenished from circulating monocytes that extravasate and differentiate locally [21]. However, lineage-tracing and fate-mapping studies over the past decade have fundamentally revised this paradigm, demonstrating that many tissue-resident macrophage populations originate prenatally from yolk sac or fetal liver progenitors and persist throughout adulthood via local self-renewal, largely independent of adult hematopoiesis [22]. Emerging evidence suggests that distinct developmental origins are linked to baseline metabolic programming, with tissue-resident macrophages exhibiting metabolically adapted, tissue-specific profiles often associated with oxidative metabolism, whereas monocyte-derived macrophages display greater metabolic flexibility in response to inflammatory and environmental cues [23].

Embryonically derived tissue-resident macrophages exhibit distinct transcriptional, epigenetic, and functional identities compared with monocyte-derived macrophages [24]. These differences include specialized cytokine expression profiles, enhanced tissue surveillance functions, long-term niche adaptation, and differential responsiveness to inflammatory and metabolic cues, which are increasingly recognized to reflect intrinsic differences in metabolic pathway utilization and bioenergetic capacity [24,25]. Resident macrophages are epigenetically programmed to maintain tissue homeostasis and immune tolerance, whereas monocyte-derived macrophages tend to display heightened inflammatory responsiveness and plasticity when exposed to environmental stressors [26]. These intrinsic differences suggest that macrophage ontogeny may precondition how cells engage glycolysis, oxidative phosphorylation, or lipid metabolism in response to tumor-associated stress.

In solid tumors such as HNSCC, the majority of TAMs are generally thought to arise from circulating monocytes that are actively recruited into TME [27]. Recent single-cell RNA sequencing of HNSCC lesions has identified multiple TAM subsets with distinct transcriptional programs, including pro-inflammatory and immunosuppressive populations, demonstrating tumor-specific heterogeneity [28]. This recruitment is mediated primarily through chemokine signaling axes such as chemokine (C-C motif) ligand 2 (CCL2)/C-C chemokine receptor 2 (CCR2) and chemokine (C-X-C motif) ligand 12 (CXCL12)/C-X-C chemokine receptor type 4 (CXCR4), which are frequently upregulated by tumor cells, stromal fibroblasts, and endothelial cells [27,29]. Once recruited, monocytes undergo rapid differentiation and reprogramming to adopt TAM-like phenotypes shaped by hypoxia, cytokine gradients, and tumor-derived metabolites, which collectively drive metabolic reprogramming toward glycolytic, lipid-dependent, and immunosuppressive states [30].

Nevertheless, tissue-resident macrophages are not eliminated during tumor development and may persist within HNSCC lesions, where they contribute to local inflammation, stromal remodeling, angiogenesis, and the establishment of immunosuppressive niches [31]. Evidence from lung, pancreatic, and colorectal cancers suggests that resident macrophages can acquire pro-tumoral functions under chronic inflammatory and metabolic stress [32,33]. In HNSCC, the relative contribution of resident versus recruited macrophages likely varies by anatomical site, tumor stage, and treatment history, influencing disease progression and therapeutic responsiveness [34]. However, precise quantification and functional dissection of these populations in HNSCC remain incompletely resolved.

Importantly, the TME imposes profound environmental pressures that differentially affect macrophage subsets [35]. Tissue-resident macrophages may possess greater metabolic flexibility or pre-programmed tolerogenic states that allow them to survive and adapt to hostile microenvironments [34,36]. In contrast, monocyte-derived TAMs often undergo extensive metabolic reprogramming to support survival and acquire specialized immune functions [34,37,38]. These ontogeny-dependent differences likely shape how macrophages respond metabolically to tumor-derived stressors, ultimately influencing their functional polarization, immunoregulatory capacity, and contribution to tumor progression.

### 2.2. Classic Innate Functions: Phagocytosis, Cytokine Secretion, and Extracellular Matrix Remodeling

Macrophages are central executors of innate immunity, performing essential roles in phagocytosis, cytokine secretion, and tissue remodeling [39]. Upon sensing pathogen-associated molecular patterns (PAMPs) or damage-associated molecular patterns (DAMPs) through pattern recognition receptors, macrophages engulf targets into phagosomes that subsequently fuse with lysosomes for enzymatic degradation [40]. This process is critical not only for microbial clearance but also for removal of apoptotic cells, necrotic debris, and transformed or stressed cells, thereby maintaining tissue integrity and immune surveillance [41].

Beyond phagocytosis, macrophages orchestrate immune responses through the secretion of a broad array of cytokines and chemokines. Pro-inflammatory mediators such as tumor necrosis factor-α (TNF-α), interleukin-1β (IL-1β), and interleukin-6 (IL-6) promote local inflammation, endothelial activation, and immune cell recruitment, whereas anti-inflammatory cytokines, including interleukin-10 (IL-10) and transforming growth factor-β (TGF-β), contribute to immune resolution and tolerance [42,43]. Chemokines such as chemokine (C-C motif) ligand 2 (CCL2) and chemokine (C-X-C motif) ligand 8 (CXCL8) recruit additional monocytes, neutrophils, and lymphocytes, shaping the cellular composition of the TME [44].

In HNSCC, distinct macrophage subsets display specialized cytokine programs. For example, SPP1^+^ macrophages exhibit elevated TNF-α and IL-1β production and have been implicated in angiogenesis, tumor cell proliferation, and recruitment of additional immunosuppressive cell populations [45]. These functional states are strongly influenced by tumor-derived cues, metabolic byproducts, and cytokines secreted by malignant and stromal cells [46,47]. TAMs also express immunomodulatory molecules such as programmed death-ligand 1 (PD-L1), IL-10, and TGF-β, directly suppressing T cell activation and dendritic cell function and facilitating immune escape [48]. This dual capacity to participate in innate defense while simultaneously enforcing immunosuppression underscores the remarkable plasticity of TAMs and their ability to integrate environmental signals to support tumor persistence.

Additionally, macrophages play a central role in remodeling the extracellular matrix (ECM). Through secretion of matrix metalloproteinases (including MMP2, MMP9, and MMP14) and cathepsins, TAMs degrade ECM components, promote tumor invasion, and facilitate metastatic dissemination [49]. These processes are energetically demanding and tightly coupled to metabolic programming [50]. Thus, innate macrophage effector functions in HNSCC are inseparable from underlying metabolic states.

### 2.3. Beyond the M1/M2 Dichotomy

The classical M1/M2 polarization framework has long been used to conceptualize macrophage activation, with M1 macrophages described as pro-inflammatory and anti-tumorigenic, and M2 macrophages characterized as anti-inflammatory, tissue-remodeling, and pro-tumoral [47]. This binary classification is largely derived from simplified in vitro stimulation paradigms, such as IFN-γ plus LPS for M1 polarization and IL-4 or IL-10 for M2 polarization [51].

However, accumulating evidence demonstrates that TAMs in vivo, particularly in HNSCC, rarely conform to discrete M1 or M2 states. Instead, they are exposed simultaneously to diverse cytokines, growth factors, hypoxia, metabolic stress, and tumor-derived metabolites, resulting in hybrid and highly plastic phenotypes [51,52]. Single-cell RNA sequencing and spatial profiling studies have identified multiple TAM subpopulations in HNSCC and other solid tumors, including SPP1^+^, APOE^+^, and MARCO^+^ macrophages, which co-express pro-inflammatory and immunosuppressive gene programs [7,53]. Conventional surface markers such as CD68, CD163, and CD206 often fail to capture this functional diversity or predict biological behavior [54]. Meta-analyses in HNSCC further reveal that expression of canonical M1 or M2 markers inconsistently correlates with patient prognosis, reinforcing the concept that macrophage function exists along a continuum rather than within a binary framework [52,55]. Macrophage states are understood to be shaped by integrated inputs from metabolic cues, cytokine milieu, epigenetic regulation, and spatial localization within the tumor [47,56].

As a result, modern immuno-oncology research is moving beyond the M1/M2 paradigm toward functional, metabolic, and spatial definitions of TAM identity. Integrating transcriptional, epigenetic, metabolomic, and spatial data offers a more accurate framework for understanding macrophage heterogeneity in HNSCC and for identifying therapeutically actionable macrophage states that drive chronic inflammation, immune suppression, and treatment resistance.

## 3. Metabolic Reprogramming of Tumour-Associated Macrophages in HNSCC

In HNSCC, tumor-associated macrophages operate within a metabolically hostile microenvironment characterized by profound hypoxia, intense nutrient competition, extracellular acidification, and accumulation of tumor-derived metabolites [57]. These conditions impose strong selective pressures that actively rewire macrophage metabolic programs, shaping their survival, functional outputs, and persistence within the tumor [58]. Rather than serving solely as a bioenergetic adaptation, metabolic reprogramming in macrophages directly regulates cytokine production, phagocytic capacity, extracellular matrix remodeling, and immunosuppressive signaling. As a result, macrophage metabolism emerges as a central determinant of innate immune function in HNSCC, integrating environmental stress signals with transcriptional and epigenetic programs that sustain chronic inflammation, dampen antitumor immunity, and promote therapeutic resistance [59].

### 3.1. Glycolysis

Classically activated macrophages are traditionally associated with enhanced aerobic glycolysis, which supports rapid ATP generation and provides intermediates for biosynthetic and inflammatory processes [60]. Glycolysis fuels the production of pro-inflammatory cytokines such as TNF-α and IL-1β, reactive nitrogen species, and the cytoskeletal remodeling required for phagocytosis [61,62,63]. In contrast, macrophages exhibiting immunoregulatory or tissue-remodeling functions rely more heavily on mitochondrial oxidative phosphorylation and fatty acid oxidation, pathways that enable sustained ATP production, mitochondrial reactive oxygen species signaling, and secretion of immunosuppressive mediators including IL-10 and TGF-β [61,63]. Within the HNSCC microenvironment, however, these metabolic programs are rarely segregated into discrete states. Instead, TAMs adopt hybrid metabolic phenotypes, simultaneously engaging glycolysis and mitochondrial respiration depending on oxygen availability, nutrient flux, and exposure to tumor-derived signals [64,65,66]. Hypoxia-inducible factor 1α (HIF-1α) acts as a central metabolic sensor in this context, stabilizing under low-oxygen conditions characteristic of head and neck tumors and driving transcription of glucose transporters and glycolytic enzymes [67]. HIF-1α signaling reinforces inflammatory gene expression while allowing macrophages to adapt to hypoxic stress [68]. This metabolic plasticity enables macrophages to rapidly rewire their functional outputs in response to dynamic tumor cues, underscoring the limitations of classical polarization models when applied to in vivo tumor settings [69]. Therapeutically, targeting glycolytic pathways has emerged as a potential strategy to modulate tumor-associated macrophage function. Inhibition of glycolysis or HIF-1α signaling may attenuate pro-inflammatory yet tumor-supportive programs and reprogram macrophages toward anti-tumor phenotypes, particularly in combination with immune checkpoint blockade.

### 3.2. Lactate Metabolism

Tumor-derived lactate represents one of the most influential metabolic signals shaping macrophage function in HNSCC [70]. Lactate is actively imported into macrophages through monocarboxylate transporters (MCTs) and functions as both a carbon source and a signaling molecule [71,72]. Lactate stabilizes HIF-1α and induces transcriptional programs associated with immunosuppressive macrophage phenotypes, including expression of arginase 1 (ARG1), vascular endothelial growth factor, and scavenger receptors [72,73]. Beyond transcriptional regulation, lactate drives histone lactylation, an epigenetic modification that directly links metabolic flux to gene expression. In HNSCC, lactate-enriched niches metabolically trap TAMs in suppressive states, reducing antigen presentation and impairing cytotoxic T cell function, which correlates with poorer patient outcomes [74]. Through this mechanism, lactate imprints long-lasting immunoregulatory programs in macrophages, reinforcing tumor-supportive functions [75,76]. In HNSCC, lactate-mediated macrophage reprogramming has broader immunological consequences that extend to adaptive immunity [77]. Lactate-induced epigenetic remodeling can promote cytokine environments that impair CD8-positive T cell cytotoxicity and favor immune escape [78]. Additional tumor-derived metabolites, including adenosine generated by ectonucleotidases, kynurenine produced through tryptophan catabolism, and bioactive lipid mediators, converge with lactate signaling to establish a metabolically conditioned immunosuppressive niche [79]. Together, these metabolites coordinate macrophage polarization toward phenotypes that support angiogenesis, dampen anti-tumor immunity, and facilitate disease progression [64]. Targeting lactate metabolism has emerged as a promising therapeutic strategy, as disruption of lactate production or transport reprograms tumor-associated macrophages away from an immunosuppressive phenotype, restores effector T cell function, and sensitizes tumors to immune checkpoint blockade [80].

### 3.3. Lipid Metabolism

Lipid metabolism constitutes another major axis of macrophage reprogramming in the tumor microenvironment [81]. TAMs frequently exhibit enhanced fatty acid uptake, lipid droplet accumulation, and reliance on fatty acid oxidation coupled to oxidative phosphorylation [82]. Lipid-rich conditions within head and neck tumors arise from tumor cell metabolism, stromal fibroblast activity, and extracellular matrix remodeling, providing abundant substrates for macrophage lipid utilization [83]. Engagement of fatty acid oxidation supports mitochondrial fitness, sustained ATP production, and activation of signaling pathways associated with immunosuppression and tissue remodeling [84]. These lipid-driven programs promote secretion of IL-10 and TGF-β, expression of immune checkpoint molecules, and extracellular matrix degradation, collectively facilitating tumor invasion and metastasis [85]. Importantly, lipid metabolism also interfaces with epigenetic regulation in macrophages [86]. Fatty acid oxidation contributes to acetyl-CoA availability, which in turn influences histone acetylation and transcriptional accessibility of immune-related genes [87]. Through this mechanism, lipid metabolism exerts durable effects on macrophage identity, reinforcing transcriptional programs linked to immune tolerance, angiogenesis, and matrix remodeling [88]. Such metabolic–epigenetic coupling highlights how bioenergetic pathways function as upstream regulators of macrophage fate rather than passive consequences of activation. Pharmacological modulation of lipid metabolism, including inhibition of fatty acid oxidation, has shown potential to reverse immunosuppressive macrophage phenotypes and limit tumor progression. These approaches may complement existing therapies by disrupting metabolic support for tumor-promoting functions [81,89].

### 3.4. Metabolic–Epigenetic Regulation

Emerging evidence further implicates specific immune-metabolic regulatory genes as integrators of lipid metabolism and innate immune signaling in HNSCC [90]. Genes such as phospholipase A2 Group IID (PLA2G2D) and tumor necrosis factor-α-induced protein 8-like 2 (TNFAIP8L2) exemplify this intersection, acting as nodes that coordinate metabolic flux with inflammatory control [91,92]. PLA2G2D encodes a secretory phospholipase involved in lipid mediator generation and immune cell recruitment, and its expression has been associated with modulation of macrophage infiltration and inflammatory tone within tumors [91,93]. TNFAIP8L2, also known as TIPE2, functions as a negative regulator of innate immune activation while directly participating in lipid transfer and metabolic homeostasis. By constraining Toll-like receptor signaling, regulating fatty acid biosynthesis, and maintaining redox balance, TIPE2 shapes macrophage metabolic programs that influence pro-versus anti-tumor activity [94,95]. Targeting these metabolic–regulatory nodes may provide additional opportunities to fine-tune macrophage function and improve therapeutic outcomes.

These findings underscore the concept that metabolism operates as an intrinsic regulator of macrophage function in HNSCC. Glycolytic flux, mitochondrial respiration, lactate signaling, and lipid metabolism converge to define macrophage phenotypes that are highly plastic, context-dependent, and deeply integrated with immune suppression and tumor progression [77,96]. This immune metabolic coupling positions tumor-associated macrophages as both drivers of disease and attractive therapeutic targets, particularly through strategies aimed at rewiring metabolic pathways, to restore anti-tumor immunity. Emerging therapeutic strategies targeting glycolysis, lactate transport, and lipid metabolism highlight the translational potential of immunometabolic interventions, particularly when integrated with immune checkpoint blockade or combination therapies in HNSCC.

## 4. Interaction of Macrophage Metabolism with Tumor Innate Immunity

The TME in HNSCC is highly metabolically restrictive due to intense competition for glucose, amino acids, fatty acids, and oxygen between proliferating cancer cells and infiltrating immune populations [19]. TAMs rely on tightly regulated metabolic programs to support phagocytosis, cytokine production, and antimicrobial activity, making them particularly vulnerable to these constraints. Limited glucose and oxygen availability impairs both glycolytic flux and mitochondrial respiration in TAMs, reducing inflammatory signaling, antigen presentation, and phagocytic capacity [97,98]. Hypoxia, arising from aberrant vascular architecture and uneven perfusion, stabilizes HIF-1α in TAMs, promoting glycolytic programs while suppressing oxidative metabolism. Although these adaptations support macrophage survival under low-oxygen conditions, they simultaneously drive expression of immunosuppressive mediators such as VEGF, arginase 1, and PD-L1, limiting effector functions [99,100].

Metabolic byproducts from tumor cells further reinforce immunosuppression. Lactate acts as both a metabolic substrate and a signaling molecule, reshaping macrophage function through NF-κB modulation and histone lactylation to promote pro-tumoral and immunoregulatory phenotypes [72]. Similarly, adenosine generated from extracellular ATP catabolism and kynurenine from tryptophan metabolism suppress macrophage activation and skew cytokine profiles toward immune tolerance [101,102]. These metabolites establish a feed-forward loop in which metabolic stress and immunosuppressive signaling mutually reinforce one another, promoting chronic inflammation while limiting innate immune responses [103,104,105,106].

The metabolic landscape of the TME is spatially heterogeneous, creating distinct niches with gradients of oxygen, nutrients, and metabolites [107]. Lactate-rich, hypoxic, and lipid-enriched regions often coincide with immune-excluded zones dominated by pro-tumoral TAMs [108,109]. In these niches, macrophages adopt alternative metabolic strategies, including increased lipid uptake and fatty acid oxidation, to sustain mitochondrial function under nutrient-poor conditions [110]. While these adaptations promote cell survival, they limit pro-inflammatory effector functions and reinforce immunosuppressive signaling [111]. Spatial multi-omic analyses integrating single-cell transcriptomics, metabolomics, and imaging reveal that TAMs in constrained niches display attenuated antigen presentation, suppressed pro-inflammatory gene expression, and enrichment of pathways linked to angiogenesis and extracellular matrix remodeling [112]. Conversely, TAMs in nutrient-rich or better-perfused regions retain greater metabolic flexibility and partial inflammatory capacity [113].

TAM metabolism both shapes and is shaped by other innate immune populations within the TME. Metabolites produced by TAMs can suppress dendritic cell (DC) activation, limiting antigen presentation and dampening T cell priming [114]. TAM-secreted cytokines recruit neutrophils and polarize them toward pro-tumoral phenotypes, thereby supporting tumor progression [115]. At the same time, natural killer (NK) cell cytotoxicity is impaired by immunosuppressive metabolites such as lactate, while myeloid-derived suppressor cells (MDSCs) expand in response to TAM metabolic signaling, further restricting T cell activity [116,117]. In HNSCC, lactate metabolism and hypoxia-associated metabolic reprogramming influence the immune microenvironment, correlating elevated lactate metabolism gene signatures with immunosuppressive features and poorer immune infiltration in tumors [118,119]. Collectively, these interactions position TAMs as central metabolic and immunoregulatory hubs, coordinating the suppression of multiple innate immune effectors and reinforcing an immunosuppressive tumor microenvironment.

Together, these findings demonstrate how tumor architecture, nutrient competition, and metabolic signaling shape innate immune landscapes. Macrophage metabolism, localization, and functional state are tightly interwoven with the metabolic topology of the tumor, collectively determining whether innate immunity restrains or supports tumor progression [120,121]. Targeting nutrient competition, hypoxia-driven signaling, or metabolite-mediated reprogramming may restore macrophage effector function and enhance the efficacy of immunotherapies in HNSCC.

## 5. Macrophage Metabolism Across Clinical and Therapeutic Contexts in HNSCC

Macrophage metabolism in HNSCC is dynamic, shaped by tumor etiology, therapeutic exposure, and disease stage, resulting in clinically meaningful heterogeneity in innate immune function [122]. Context-dependent metabolic programs in TAMs help explain divergent immune landscapes, variable responses to immunotherapy, and patterns of resistance not fully captured by conventional immune phenotyping [17]. Understanding these programs is therefore essential for translating immunometabolic insights into patient-specific therapeutic strategies.

Human papillomavirus (HPV)-positive and -negative HNSCC represent biologically distinct diseases with divergent immune and metabolic landscapes [123]. Single-cell profiling of HPV-positive and HPV-negative HNSCC has revealed distinct macrophage subset compositions and functional signatures associated with etiology-specific immune phenotypes [28], while metabolic analyses of tumor cells indicate differences in glycolytic and energy pathway utilization that may influence the surrounding immune microenvironment [124]. HPV-positive tumors generally display higher immune infiltration, increased CD8^+^ T cell density, and enhanced responsiveness to immune checkpoint blockade, reflecting microenvironments that favor macrophage plasticity and pro-inflammatory function [125,126,127,128,129,130]. In contrast, HPV-negative tumors, often associated with tobacco and alcohol exposure, are characterized by pronounced hypoxia, elevated glycolysis, and lactate accumulation, which stabilize HIF-1α in TAMs and drive immunosuppressive programs marked by arginase-1 expression, PD-L1 upregulation, and impaired antigen presentation [127,128]. Emerging evidence suggests that TAMs in HPV-positive tumors adopt intermediate metabolic states, integrating glycolysis with mitochondrial oxidative metabolism to support immune cross-talk, whereas lactate-rich niches in HPV-negative tumors trap TAMs in suppressive states, limiting their capacity to promote antitumor immunity and reducing immunotherapy responsiveness [129,130,131,132,133,134].

Standard therapies—including radiation, platinum-based chemotherapy, and immune checkpoint blockade—profoundly remodel the metabolic architecture of the TME, affecting macrophage function [135]. Radiation induces vascular damage and hypoxia, enhancing glycolysis and lactate accumulation, while promoting TAM HIF-1α signaling, lipid uptake, and oxidative metabolism that supports tissue remodeling rather than tumor clearance [136,137]. Chemotherapy induces immunogenic cell death while releasing metabolites and damage-associated molecular patterns that skew macrophage metabolism [138]. Immune checkpoint blockade restores T cell glycolysis and effector function, indirectly reshaping tumor metabolic dominance, with compensatory TAM shifts toward oxidative or lipid-dependent programs in nutrient-limited settings [139,140].

Macrophage metabolic states also evolve during tumor progression, reflecting cumulative changes in nutrient availability, oxygen tension, and stromal remodeling [141]. Early lesions with preserved perfusion support glycolysis-driven inflammatory programs that can restrain tumor growth [38], whereas advanced tumors impose selective pressures favoring TAMs with enhanced mitochondrial fitness, lipid utilization, and stress tolerance, reinforcing immunosuppressive phenotypes [38,111,142].

This context-dependent metabolism has implications for biomarker development. Static immune markers, such as macrophage density or M1/M2-associated gene expression, fail to capture TAM functional states in metabolically constrained environments [64]. Metabolism-based biomarkers, including glycolytic activity, fatty acid oxidation, lipid species, or macrophage-specific regulators (e.g., PLA2G2D, TNFAIP8L2), offer dynamic readouts of immune competence, predicting immunotherapy response, recurrence risk, and resistance [92,93,143,144,145]. Longitudinal monitoring using circulating metabolites, exosomal cargo, or transcriptional signatures from minimally invasive sampling could inform adaptive treatment strategies and integrate TAM metabolism as both a therapeutic target and a clinically actionable indicator of tumor-immune equilibrium [146,147,148].

## 6. Clinical Implications and Therapeutic Opportunities

**Figure 2:**
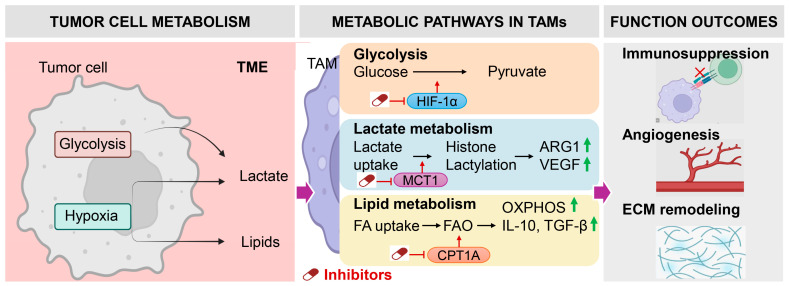
Metabolic regulation of TAMs in HNSCC. Hypoxic tumor cells enhance glycolysis and lipid release, leading to lactate and fatty acid accumulation. In TAMs, lactate uptake via monocarboxylate transporter 1 (MCT1) drives histone lactylation and induces ARG1 and VEGF, while fatty acids fuel carnitine palmitoyltransferase 1A (CPT1A)-dependent fatty acid oxidation (FAO) to sustain oxidative phosphorylation (OXPHOS) and IL-10/TGF-β production. HIF-1α further promotes glycolytic reprogramming. These pathways collectively drive immunosuppression, angiogenesis, and extracellular matrix remodeling, and represent therapeutic targets.

The growing recognition that tumor-associated macrophage function is governed by metabolic programming has opened new therapeutic avenues for reshaping the immune landscape of HNSCC (Figure 2) [149]. Unlike irreversible genetic alterations in tumor cells, macrophage metabolic states are highly plastic, making them particularly amenable to therapeutic reprogramming. Targeting metabolic pathways that sustain immunosuppressive macrophage phenotypes offers a strategy to restore pro-inflammatory, anti-tumor innate immune activity while simultaneously enhancing adaptive immune responses [150].

While glycolysis supports pro-inflammatory cytokine production under acute immune activation, chronic tumor-driven glycolysis and excessive lactate accumulation reinforce immunosuppressive macrophage states [77,151]. Pharmacologic inhibition of key glycolytic regulators or blockade of lactate production has been shown in preclinical models to reduce extracellular lactate levels and attenuate pro-tumoral macrophage polarization [70,152]. Importantly, limiting lactate signaling not only restores macrophage inflammatory capacity but also alleviates lactate-mediated suppression of cytotoxic T cells and dendritic cells, thereby promoting coordinated innate and adaptive anti-tumor immunity [153].

Lipid metabolism and fatty acid oxidation play an equally critical role in sustaining immunosuppressive macrophage phenotypes within the tumor microenvironment [154]. Enhanced fatty acid uptake and mitochondrial oxidative metabolism support long-term macrophage survival under nutrient-poor conditions while activating anti-inflammatory signaling pathways such as Janus kinase 1 (JAK1)-signal transducer and activator of transcription 6 (STAT6) [66]. Inhibition of fatty acid oxidation, particularly through targeting carnitine palmitoyltransferase 1A (CPT1A), disrupts this metabolic dependency and shifts macrophages toward pro-inflammatory states characterized by increased production of TNF-α and IL-12 [155,156]. Preclinical studies indicate that modulation of lipid metabolism, particularly inhibition of fatty acid oxidation, can enhance effector T cell function and improve responses to immunomodulatory therapies, highlighting the translational potential of targeting metabolic pathways within the tumor microenvironment [157].

Hypoxia-driven metabolic adaptation further contributes to macrophage-mediated immune suppression. Stabilization of hypoxia-inducible factor 1α under low oxygen conditions promotes glycolytic flux while inducing expression of immunosuppressive mediators such as arginase 1, vascular endothelial growth factor, and PD-L1 [158]. Experimental modulation of hypoxia-inducible factor signaling has been shown to regulate macrophage metabolic state and function, with reduced HIF activity enhancing oxygen-dependent processes such as mitochondrial reactive oxygen species production and phagocytic capacity, while attenuating angiogenic and immune-suppressive programs associated with hypoxic environments [159]. These interventions enhance innate immune surveillance and improve tumor control in preclinical settings.

Beyond glucose and oxygen metabolism, therapeutic manipulation of lipid handling pathways provides additional opportunities to reprogram macrophage function. Interfering with fatty acid uptake or lipid droplet formation in tumor-associated macrophages can disrupt the metabolic circuits that support immunosuppressive macrophage states and tumor progression [154]. Conversely, activation of metabolic sensors such as AMP-activated protein kinase (AMPK) promotes energy homeostasis and influences macrophage metabolic and functional states, supporting inflammatory and immune activities that can enhance antigen presentation and crosstalk with T cells to reinforce immune-mediated tumor control [160].

Metabolic reprogramming of macrophages has particularly important implications for overcoming resistance to immunotherapy. Immune checkpoint inhibitors targeting the programmed cell death protein 1/programmed death-ligand 1 (PD-1/PD-L1) axis have transformed the treatment landscape of HNSCC, yet a substantial proportion of patients exhibit primary or acquired resistance and derive limited benefit from these therapies, underscoring the need for improved strategies [161,162,163]. A central driver of therapeutic failure is the metabolically suppressive tumor microenvironment, characterized by nutrient depletion, hypoxia, and the accumulation of inhibitory metabolites that impair both innate and adaptive immune effector functions, thereby limiting responses to immune checkpoint blockade [164,165,166]. TAMs play a pivotal role in enforcing this suppressive state by limiting T-cell activation, restricting immune infiltration, and sustaining chronic inflammation within the tumor microenvironment, thereby contributing to immune evasion and resistance to immunotherapy [167,168,169].

Combining metabolic interventions with immune checkpoint blockade offers a rational strategy to overcome these barriers. Preclinical evidence indicates that metabolic interventions targeting lactate signaling, fatty acid oxidation, or hypoxia-associated pathways can alleviate macrophage-mediated immunosuppression, enhance T-cell cytotoxicity, and improve responsiveness to PD-1/PD-L1 immune checkpoint blockade, including strategies to reduce lactate levels with monocarboxylate transporter 1 (MCT1) inhibitors to sensitize resistant tumors [170,171]. Activation of metabolic regulators such as AMP-activated protein kinase biases macrophages away from suppressive phenotypes and toward metabolic states that support immune surveillance and anti-tumor immunity [160]. Metabolism-based combination strategies extend beyond checkpoint inhibitors to include radiation therapy, cytotoxic chemotherapy, and oncolytic viruses, as these modalities can induce immunogenic cell death accompanied by transient metabolic shifts that favor innate immune activation and enhance anti-tumor immune responses [172,173,174]. When synchronized with macrophage-targeted metabolic modulators, approaches that reprogram the metabolic state of tumor-associated macrophages and other immune cells have the potential to remodel the tumor microenvironment toward a more immunogenic “hot” phenotype characterized by enhanced immune infiltration and anti-tumor activity, presenting a promising avenue to improve therapeutic outcomes [175,176].

Collectively, targeting macrophage metabolism represents a versatile and clinically actionable strategy to reshape the tumor microenvironment, overcome innate and adaptive immune suppression, and enhance the efficacy of existing and emerging therapies in HNSCC.

## 7. Future Directions

Understanding and therapeutically manipulating macrophage metabolism in HNSCC remains a rapidly evolving frontier. Recent advances in single-cell and spatial omics, integrated with computational analyses, now enable high-resolution interrogation of immune and metabolic programs across complex tissue microenvironments, uncovering previously unrecognized cellular and spatial heterogeneity that underlies chronic inflammation, establishes immunosuppressive niches, and contributes to therapeutic resistance [177,178,179,180]. Future progress in this field will depend on integrating high-dimensional metabolic profiling with spatial context, functional validation, and clinically actionable biomarker development [181].

Single-cell metabolic profiling represents a critical next step in resolving the functional diversity of tumor-associated macrophages. Conventional bulk transcriptomic and metabolomic analyses average signals across heterogeneous cellular populations, thereby obscuring rare or transitional macrophage states that may exert disproportionate influence on tumor progression, whereas integrative single-cell approaches can resolve these functionally distinct subpopulations [182]. Emerging methods now enable direct measurement of gene expression and metabolite abundance in individual cells, facilitating the assessment of metabolic pathway utilization at single-cell resolution [183]. Recent single-cell analyses in HNSCC further demonstrate that TAMs exist in metabolically heterogeneous and plastic states linked to diverse functional programs, including pro-inflammatory activity, immune suppression, tissue remodeling, and angiogenesis, highlighting tumor-specific metabolic heterogeneity within the microenvironment [184]. Early studies in solid tumors also show that macrophage subsets segregate according to dominant metabolic programs, such as glycolysis, fatty acid oxidation, or mitochondrial respiration, with these states closely associated with cytokine production, antigen presentation capacity, and responsiveness to immunotherapy [185]. Extending these approaches in HNSCC will clarify how metabolic plasticity relates to disease stage, treatment exposure, and clinical outcomes, while identifying metabolic dependencies that can be selectively targeted without disrupting systemic immunity.

While single-cell profiling captures cellular heterogeneity, it inherently disrupts spatial information, a limitation that spatial transcriptomic approaches address by preserving tissue architecture and enabling analysis of metabolic gradients and cellular niches characteristic of solid tumors [186]. Integrating spatial transcriptomics with metabolic phenotyping offers a powerful strategy to overcome this limitation. Technologies combining spatially resolved gene expression with imaging mass spectrometry and multiplexed metabolite detection now enable visualization of macrophage metabolic states within intact tissue architecture [187,188]. In HNSCC, such approaches can define how hypoxic zones, lactate-enriched regions, and lipid-rich stromal compartments shape macrophage polarization and innate immune function in situ. Spatial analyses in other tumor types demonstrate that macrophages localized to hypoxic, metabolite-rich niches exhibit impaired antigen presentation and heightened immunosuppressive signaling, whereas macrophages in better-perfused regions retain inflammatory and immune-supportive programs [189]. These spatially constrained metabolic and immune phenotypes strongly predict immune exclusion and response to immunotherapy, underscoring the need to incorporate anatomical context into immunometabolic models [180]. Applying integrated spatial-metabolic profiling to HNSCC will enable identification of niche-specific vulnerabilities and guide interventions tailored not only to metabolic state but also to tumor geography.

As macrophage metabolism emerges as a central regulator of innate immune function, the development of metabolism-based biomarkers represents an important translational objective. Pan-cancer analyses already suggest that such metabolic signatures correlate with macrophage infiltration, immune suppression, and patient survival, and preliminary evidence indicates their potential utility in predicting response to immune checkpoint blockade and metabolic therapies [190]. In HNSCC, systematic validation of these markers could enable improved risk stratification and personalized treatment selection. Beyond tissue-based markers, circulating metabolic indicators offer opportunities for minimally invasive monitoring. Plasma metabolites, lipid profiles, and exosome-associated cargo can reflect macrophage metabolic states and provide dynamic, non-invasive readouts of tumor immune status and potential therapeutic response, as circulating exosomal lipidomic and metabolomic signatures differ between cancer patients and healthy controls [191]. Integration of multi-omic plasma profiling with machine learning approaches may yield composite innate immune–metabolic biomarkers capable of tracking macrophage reprogramming over time, anticipating therapeutic resistance, and guiding adaptive treatment strategies [192].

Future progress will also depend on experimental systems and computational frameworks that enable functional validation and predictive modeling. In vivo metabolic tracing using stable isotope-labeled nutrients enables assessment of metabolic fluxes within intact tumors and the surrounding microenvironment [193], while three-dimensional tumor–macrophage organoid co-culture systems allow mechanistic interrogation of macrophage–tumor interactions in controlled settings [194]. Coupled with genetically engineered mouse models and related in vivo systems, these approaches can be used to test causal relationships between metabolic pathways and macrophage function [193]. At the same time, computational integration of single-cell, spatial, and circulating data will facilitate systems-level modeling of immunometabolic networks, enabling identification of synthetic lethal interactions and rational combination therapies [195]. Such integrative approaches are likely to accelerate translation from descriptive immunometabolism to mechanism-driven therapeutic intervention.

Collectively, advances in single-cell and spatial profiling, biomarker discovery, and integrative modeling promise to transform understanding of macrophage metabolism in HNSCC. By resolving metabolic heterogeneity in space and time and linking it to functional immune outcomes, future studies will lay the foundation for metabolism-guided immunotherapies capable of reshaping the innate immune landscape and improving patient outcomes.

## 8. Conclusions

Macrophage metabolism is a central determinant of innate immunity, tumor progression, and therapeutic responsiveness in HNSCC. Advanced single-cell and spatial technologies, combined with mechanistic studies of metabolic reprogramming, offer unprecedented opportunities to define TAM heterogeneity, identify metabolic vulnerabilities, and guide novel therapeutic strategies. Targeting metabolic pathways in TAMs may overcome immunosuppression, enhance immunotherapy efficacy, and improve patient survival, marking a promising frontier in HNSCC research.

## Data Availability

No new data were created or analyzed in this study.
